# PDA Stenting for Ductal-Dependent Cyanotic Congenital Heart Disease: History and View From 10,000 Feet

**DOI:** 10.1007/s00246-024-03714-3

**Published:** 2024-11-25

**Authors:** John W. Moore

**Affiliations:** Emeritus UC San Diego School of Medicine, 9640 Deer Trail Drive, San Diego, CA 92127 USA

**Keywords:** Blalock-Taussig-Thomas shunt, PDA stent, Cyanotic congenital heart disease

## Abstract

This article provides a historical review and a current perspective on the procedures used to palliate cyanosis in ductal-dependent infants. Eighty years ago, Helen Taussig, Alfred Blalock and Vivien Thomas developed the first effective treatment. The Blalock-Taussig-Thomas (BTT) shunt is the historical predecessor of both the contemporary modified BTT shunt and interventional stenting of the Patent Ductus Arteriosus (PDA). The surgical shunt was firmly established therapy before catheterization was born, and PDA stenting was not possible until the technologies designed to address coronary heart disease were developed. The momentum of long-established surgical therapy inhibited clinical development of PDA stenting. Nevertheless, available clinical outcomes, though limited, appear to favor PDA stenting, and first-line therapy may be shifting from the modified BTT shunt to PDA stenting. More definitive data should arise from a randomized controlled trial.

## Introduction

According to the Centers for Disease Control and Prevention (CDC), life expectancy last year in the United States was 77.5 years. This is an astounding figure if you consider that life expectancy one hundred years ago was only about 50 years. Profound improvement in infant mortality (which dropped from 165 deaths per 1000 births in 1900 to about 7 in 2024) accounts for much of the gain. Clean drinking water, good sanitation and the development of vaccines and antibiotics have enabled many more children to survive infancy [1]. Buried within this data is also an increment of improvement related to the development of congenital heart surgery. Survival of infants with cyanotic congenital heart disease was less than 10% before 1940. The advent of congenital heart surgery completely reversed this sad statistic. Today more than 90% of such infants can expect to survive infancy and enter adulthood. [2]

The first major step in this reversal was taken by the unlikely collaboration of Helen Taussig, Alfred Blalock, and Vivien Thomas. This paper tells the story of these pioneers who overcame personal hardships and discrimination, and through imagination, commitment, and persistence, developed a surgical procedure which entirely changed the outlook for patients with cyanotic congenital heart disease. It also carries the story forward to more recent times when another generation of cardiologists and surgeons applied new technologies to develop a less-invasive alternative procedure which eventually achieved acceptance.

## Taussig, Blalock, and Thomas

Helen Taussig was born in Cambridge, Massachusetts, in 1898. As a child, she experienced significant hardships: Her mother died from tuberculosis when she was 11 years old. She was dyslexic and became partially deaf (from an ear infection) after age 11. Consequently, she was a mediocre student. Nonetheless, she powered through school with the help of her father’s tutoring and was admitted to Radcliffe College. After two years she transferred to Berkeley where Taussig completed her undergraduate degree. Determined to go to medical school, she returned to Cambridge only to find that neither Harvard nor Boston University admitted women into their MD programs. She eventually enrolled at Johns Hopkins and was awarded the MD degree in 1927. She completed pediatric training and a fellowship year studying cardiology. Subsequently in 1930, she was assigned to run the pediatric rheumatic heart clinic. This quickly expanded into a pediatric heart clinic accepting many patients with congenital heart disease.

Because only supportive treatment was available, the cyanotic children drew her attention [3, 4]. Diagnostic methods at this time were limited to examination, fluoroscopy, and ECG; but by 1936, Maude Abott had fully described most cyanotic congenital lesions from pathological specimens [5]. Taussig became skilled at diagnosing cyanotic patients with inadequate pulmonary blood flow and particularly those with Tetralogy of Fallot. She established a large practice of cyanotic children. Taussig reasoned that the major blood vessels “could be altered” to provide more blood flow to the lungs. In 1939, Robert Gross at Harvard reported the first successful ligation of a Patent Ductus Arteriosus. Because of his interest and prominence in ductal surgery, Taussig approached Gross and requested that he consider creating an “artificial ductus” to treat cyanotic children. Gross declined [3, 4]. Fortunately, his refusal delayed Taussig’s undertaking, but did not end it.

Alfred Blalock was born in Culloden, Georgia in 1899. He attended the University of Georgia and Johns Hopkins Medical School. He distinguished himself in surgical courses and decided to pursue this path for a career. However, he was not accepted into the Hopkins surgical residency. In 1925, Blalock moved to Vanderbilt, where he was the first surgical resident in that institution. He completed training and remained on the Vanderbilt surgical faculty until 1941. Early in his training he developed a strong interest in surgical research. He spent many hours in the animal laboratory. As a junior attending surgeon, he was fortunate to hire Vivien Thomas to assist in his laboratory. [6]

Vivien Thomas was an African American born in New Iberia, Louisiana, in 1910 during the Jim Crow era. Thomas attended segregated schools in Nashville and was an excellent student. In 1929, with the ultimate goal of becoming a physician, he entered Tennessee Agricultural and Industrial State College, one of the few institutions which would admit African American students. He was forced to drop out after only one semester because his family lost their savings in the Great Depression, and for financial reasons he was never able to complete his formal education. Thomas secured a job as an assistant in Blalock’s laboratory at Vanderbilt. [7]

The collaboration of Blalock and Thomas began in 1930. Blalock quickly appreciated Thomas’ dexterity, intelligence, and interest in medicine. He awarded Thomas increasing independence. Within a short time, Blalock would frame a problem and ask Thomas to solve it using an appropriate animal model. This suited Thomas very well, and their relationship thrived. Together while at Vanderbilt, they elucidated the mechanism of hemorrhagic shock as caused by a loss of blood. Their work in this area supported the wide-spread use of blood plasma on the battlefield and is credited with saving thousands of lives during World War II. In addition, in 1938, Thomas devised a surgical procedure consisting of connecting the left subclavian artery to the left pulmonary artery. The goal was to create a model of pulmonary hypertension. The model failed because the animals became acutely over-circulated. However, this procedure provided the basis for their groundbreaking clinical work to come a few years later. [7]

In 1941, Blalock was recruited to Johns Hopkins to lead the Division of Surgery. As part of his recruitment, Blalock insisted that Thomas also be offered a position in the animal laboratory. Thomas came – but was paid as a janitor because in those days African Americans were not allowed to fill professional positions at Johns Hopkins. Blalock and Thomas continued their collaboration. In 1942, Blalock performed the first PDA ligation at John’s Hopkins. The procedure was witnessed by Taussig, and she approached Blalock as she had previously approached Gross. Blalock was interested, but he insisted that an animal model be created before he would attempt the operation on a cyanotic child. He assigned the project to Thomas. [6]

Thomas labored the next year to create a reliable dog model of cyanosis. He operated on almost 200 animals. He achieved success in several dogs by anastomosing a pulmonary vein to a pulmonary artery and resecting part of the lung. After recovering, the dogs had clinical cyanosis. Subsequently, Thomas created the first shunts (end-to-side anastomosis of the left subclavian artery to the left pulmonary artery) to alleviate the cyanosis. Several of the dogs were long-term survivors. The most famous was “Ana,” who became the lab mascot and whose portrait was displayed on the laboratory wall for many years. Blalock performed one of the operations and determined that he was prepared to attempt this procedure in humans. [8]

## The Blalock-Taussig-Thomas Shunt

The first human procedure was performed by Blalock and his surgical team on November 29, 1944. Thomas was present and guided Blalock through the procedure with numerous suggestions. Taussig was also in the room assisting in general patient care. The patient was a 4.5-kg, 15-month-old girl with severe Tetralogy of Fallot. The left subclavian artery was anastomosed to the left pulmonary artery. The procedure was difficult because of small vessel size, a highly vascular mediastinum and difficult visibility. The patient’s cyanosis was improved after the procedure, and she left the hospital two weeks later. After two months, she suffered a recurrence of severe cyanosis. A second shunt was attempted on the right side but was unsuccessful.

Although the first procedure was problematic, Blalock was sufficiently encouraged to continue. He and Taussig reported 3 successful cases in the Journal of the American Medical Association, six months later [9]. Reports of the shunt palliating critically cyanotic children spread rapidly through both professional and lay channels. John’s Hopkins rapidly became the epicenter of performing and teaching shunt procedures. By the early 1960’s the procedure had been “modified” with use of a tube graft such that the courses of the great vessels were no longer altered. Williams et al. reported the Johns Hopkins six-decade experience with the shunt (from 1944 to 2006). Two thousand and sixteen shunts were placed by 28 surgeons in 1880 patients, 72% with the diagnosis of Tetralogy of Fallot. Overall operative mortality was 14% for the early shunt procedures. This improved slightly over the decades, but improvement did not reach statistical significance [10]. The Hopkins experience became the standard of care.

By the early 1950’s other major cardiac centers began using the shunt to palliate cyanotic patients. In addition, repair of cyanotic patients with Tetralogy of Fallot became an alternative option for some patients. C. Walton Lillehei performed the first repairs in 1954 using human cross-circulation (parent and child). After the heart–lung machine became available in 1955, it was used to enable open-heart repairs [11]. As techniques improved, surgeons were able to repair smaller patients with Tetralogy. Consequently, palliative shunts were increasingly reserved for younger patients with smaller pulmonary arteries, single ventricle, or other risk factors.

Near-contemporary studies continue to report significant risks of modified BTT shunts. In 2011 using Society for Thoracic Surgery data gleaned from 86 centers, Petrucci et al. reported short-term results in 1,273 patients undergoing shunts. Discharge mortality was 7.2% and composite morbidity (defined as requirement for reoperation, use of ECMO or low cardiac output) was 13.1% [12]. In 2016, Dorobantu et al. reported outcomes of shunts performed between 2000 and 2013 and registered with the United Kingdom’s National Institute for Cardiovascular Outcomes Research. Thirty-day mortality for all procedures performed from 2007 to 2012 was 9.8%. Overall, the data showed a significant trend of increasing shunt mortality over the interval reported [13]. This trend was attributed to an enlarging percentage of high-risk patients. Thus, even in the most recent large reports, the surgical risk of Blalock–Taussig–Thomas Shunts remains significant. This fact has been foundational for the development of an interventional alternative.

## Background of the Interventional “Shunt”

All of cardiac catheterization and interventional cardiology developed after the surgical shunt procedure had become established clinical practice.

In 1929, Werner Forssmann first performed a right heart catheterization on himself using a urological catheter in Berlin. He documented the procedure with an x-ray but did no follow-up studies. Inspired by Forssman’s demonstration, Andre Cournand and Dickinson Richards established the first diagnostic cardiac catheterization laboratory in 1945 at Bellevue Hospital. They performed and reported hemodynamic studies on patients with various congenital heart defects. In 1956, Forssmann, Cournard and Richards were awarded the Nobel Prize in Medicine for “their discoveries concerning heart catheterization and pathological changes in the circulatory system.” [14] In 1958, F. Mason Sones initiated the era of diagnostic imaging by performing the first selective coronary angiogram [15]. Lionel Bargeron brought imaging to congenital heart disease by developing axial cineangiography in the 1970’s [16].^.^

Interventional catheterization should be traced to Charles Dotter, a radiologist who performed the first percutaneous peripheral vessel dilation using metal dilators in 1964 [17]. Andreas Gruntzig developed the first contemporary-looking angioplasty balloon catheter and performed the first coronary angioplasty in 1974 [18]. Modern stents were designed and developed by Julio Palmaz who first patented them in 1985 [19]. Stent technology was applied to congenital heart disease by Charles Mullins who reported “first use” in pulmonary arteries and systemic veins in 1988 [20]. Palmaz collaborated with Richard Schatz in downsizing the stent to be appropriate for use in coronary arteries. The Palmaz-Schatz coronary stent received FDA approval in 1994. [21]

With the development of axial cine-angiography and coronary-sized angioplasty catheters and stents, the fundamental tools and devices required for PDA stenting were in place.

## Development of Patent Ductus Arteriosus (PDA) Stenting

In the final decades of the twentieth century, innovation in pediatric interventional cardiology was exploding. Motivated by the conviction that “non-invasive” procedures potentially offered major patient benefits, pioneers including Raskind, Mullins, Lock, and their protegees developed procedures to treat numerous congenital heart lesions previously addressed by open-chest or open-heart surgery. These included balloon atrial septostomy, pulmonary and aortic valvuloplasty, coarctation, and pulmonary artery angioplasty and stenting, and closure of patent ductus arteriosus and septal defects. Because industry partners were few and budgets for pediatric devices or device applications were very limited, many of the procedures relied on use or modification of devices and technologies designed for coronary or peripheral vessels.

In this environment, as if to answer Helen Taussig’s request for an “artificial ductus” from decades earlier, Coe and Moore reported the first animal PDA stent studies in 1991. These newborn lamb studies demonstrated a high percentage of procedure success and modest duration of stent patency (up to 10 weeks). Importantly, in the lamb model, the ductus arteriosus has a short, straight course between the great vessels. This made it relatively simple to implant the short, stiff Palmaz-Schatz coronary stent and to achieve persistent ductal patency [22, 23]. Successful lamb studies encouraged early efforts to stent PDAs in cyanotic infants. Initial human reports appeared soon after publication of the lamb studies. Gibbs reported mortalities in both of his team’s attempts as early as 1992 [24]. After other scattered early attempts also suggested prohibitive risk, a hiatus of nearly a decade followed.

During this period, there were major advances in coronary stents. Newly designed stents were longer and more flexible. These stents were capable of traversing and molding more complex vessel curves. New coronary guidewires developed with both floppier and stiffer options. Microcatheters able to guide and to track coronary guidewires also became available. In addition, digital imaging was developed and was applied to small blood vessels. All these technical advances, designed for coronary angioplasty and stenting, were eventually applied to PDA stenting.

More than a decade later much improved results were reported from a developing country. In 2004, Alwi and colleagues in Malaysia reported a series of 56 unselected cyanotic infants who underwent attempted PDA stenting. Procedure success was 91% and procedure-related mortality was 5.9%. Freedom from reintervention was 89% after six months [25]. Their results were comparable to surgical shunt results. Alwi’s experience created a rebirth of interest in PDA stenting among interventionalists in first world cardiac centers.

However, there were significant obstacles. In the early 2000’s, centers with excellent cardiac surgery had very established practices of palliating cyanotic infants using the modified BTT shunt. Surgical results were predictable and improvements in cardiac intensive care seemed to be making surgery safer. In addition, the stent procedure required “off-label” use of coronary technologies, as these stents were not designed or FDA-approved for this specific indication. Modifications of these technologies were operator-specific, and consensus about procedural details had not been established. Even the most appropriate vascular access sites were debated. Furthermore, there was little clinical data supporting this procedure as an alternative to traditional surgical therapy. Given these circumstances, it is amazing that PDA stenting was eventually developed to the point of challenging the modified BTT Shunt.

It is not surprising that criteria for initial PDA stent cases in centers with excellent cardiac surgery were highly selective. Early cases often had uncomplicated anatomy such as the ductal morphology usually found in patients having Pulmonary Atresia with Intact Ventricular Septum. Because dual blood supply provided sufficient safety margin for non-emergent surgical referral in cases of stent failure, operators also often required dual pulmonary blood supply as in Pulmonary Atresia patients after valve perforation and valvuloplasty. Notwithstanding the perceived low risk of such patients, early outcomes were uncertain, and durability of outcomes was unknown. Furthermore, since many patients would eventually require repair or additional palliation, surgeons were not always comfortable with how stented patients would be managed and whether pulmonary arteries would grow or become distorted.

Early favorable procedural outcomes were sometimes dismissed or not considered comparable to surgical results. Stent reports were limited to specific diagnoses of patients (e.g., Pulmonary Atresia) or comprised multi-center “Case Control” studies. Nevertheless, outcomes generally favored stenting with mortalities in stent patients in the 6–8% range and in shunt cases 10–12%. Lengths of hospital stay were also shorter among stent patients. The main disadvantage seemed to be a need for earlier reinterventions (usually stent re-dilations) [26–29].^.^

Given accumulating favorable data, coupled with considerable PDA stent experience and an imaginative, supportive, and closely aligned surgical team, the group at Rady Children’s Hospital in San Diego took a “leap of faith” late in 2017. PDA Stenting was elevated to first-line therapy for palliation of all ductal-dependent cyanotic newborns. In the initial 2021 report, Ratnayaka compared outcomes for the period in which all patients had ductal stenting (2018–2020) to an earlier period (2013–2017) in which only “selected” patients underwent stenting. They found no difference in mortality, treatment failures, complications, or reinterventions between the eras. Furthermore, there were clear advantages in hospital and CVICU length-of-stays noted in the stent-only era. [30]

## PDA Stenting in the Current Era

Important additional data has emerged regarding PDA Stenting. Ganta reported on interstage care and Stage 2 palliation among functional single ventricle patients undergoing Stage 1 palliation with either surgical shunts or PDA stents. Survival to Stage 2 was similar between groups, and both groups had no Stage 2 mortality. However, CVICU time and hospital length of stay were shorter in the stent group. Furthermore, neurodevelopmental outcomes were also better among the stent patients [31]. El Said confirmed these neurodevelopmental findings in larger groups of patients. [32]

It is reasonable to assert that other centers have elevated PDA Stenting to first-line therapy [31]. However, in the current era, when there are alternative treatment options, controlled clinical trials provide the best comparative data. The Compass Trial (Clinical Trials.gov ID: NCT05268094) “Comparison of Methods of Pulmonary Blood Flow Augmentation in Neonates: Shunt Versus Stent” is underway. It is a prospective, multi-center, randomized trial comparing outcomes in patient groups having either surgical shunts or PDA stents. Patients must be followed for one year after their procedures, and study completion is estimated to be late in 2026. This trial may provide further data validating PDA Stenting. Finally, at least one MedTech company is developing a new self-expanding device, purpose-built for PDA stenting. This device may offer further advantages over current “off-label” use of borrowed technology. [33]

## Conclusion

The story of PDA stenting is a story like many others in the history of medicine and science. New ideas and technologies are resisted by established beliefs and methods. Recognition of beneficial change comes slowly, and change is introduced gradually and incrementally.

Eighty years later, it is apparent that the original Blalock-Taussig-Thomas shunt is the antecedent of modern methods for palliating ductal-dependent cyanotic newborns. The BTT shunt became established therapy in the 1950’s and with a surgical modification continued as the principle available therapy for at least 60 years. Beginning in the early 2000’s, this established surgical procedure has been challenged by an interventional alternative. As of this writing, cyanotic newborn patients may be offered either a surgical shunt or an interventional procedure to stent the ductus arteriosus. Based on equivalent or lower risks, shorter inpatient stays, lower requirements for intensive care, as well as lesser degrees of patient and family trauma, stenting appears to be gaining favor.

As a final note, the nomenclature of the shunt is worth highlighting. In the initial reports from Blalock and Taussig, there was no mention of Thomas. The eponym, Blalock–Taussig Shunt emerged in the 1970’s after deLeval used it to report his case series [34]. This shorthand was accepted and was subsequently widely used. The substantial contributions of Thomas were not recognized until 2003, when Brogan proposed changing the eponym to Blalock–Taussig–Thomas Shunt [35].

## Competing Interests

The authors declare no competing interests.

## Data Availability

No datasets were generated or analysed during the current study.
